# Use of medicines by patients of the primary health care of the Brazilian Unified Health System

**DOI:** 10.11606/S1518-8787.2017051007144

**Published:** 2017-09-22

**Authors:** Clarisse Melo Franco Neves Costa, Micheline Rosa Silveira, Francisco de Assis Acurcio, Augusto Afonso Guerra, Ione Aquemi Guibu, Karen Sarmento Costa, Margô Gomes de Oliveira Karnikowski, Orlando Mario Soeiro, Silvana Nair Leite, Ediná Alves Costa, Renata Cristina Rezende Macedo do Nascimento, Vânia Eloísa de Araújo, Juliana Álvares

**Affiliations:** I Programa de Pós-Graduação em Medicamentos e Assistência Farmacêutica. Faculdade de Farmácia. Universidade Federal de Minas Gerais. Belo Horizonte, MG, Brasil; II Departamento de Farmácia Social. Faculdade de Farmácia. Universidade Federal de Minas Gerais. Belo Horizonte, MG, Brasil; III Faculdade de Ciências Médicas. Santa Casa de São Paulo. São Paulo, SP, Brasil; IV Núcleo de Estudos de Políticas Públicas. Universidade Estadual de Campinas. Campinas, SP, Brasil; V Programa de Pós-Graduação em Saúde Coletiva. Departamento de Saúde Coletiva. Faculdade de Ciências Médicas. Universidade Estadual de Campinas. Campinas, SP, Brasil; VI Programa de Pós-Graduação em Epidemiologia. Faculdade de Medicina. Universidade Federal do Rio Grande do Sul. Porto Alegre, RS, Brasil; VII Faculdade de Ceilândia. Universidade de Brasília. Brasília, DF, Brasil; VIII Faculdade de Ciências Farmacêuticas. Pontifícia Universidade Católica de Campinas. Campinas, SP, Brasil; IX Departamento de Ciências Farmacêuticas. Universidade Federal de Santa Catarina, Florianópolis, SC, Brasil; X Instituto de Saúde Coletiva. Universidade Federal da Bahia. Salvador, BA, Brasil; XI Instituto de Ciências Biológicas da Saúde. Pontifícia Universidade Católica de Minas Gerais. Belo Horizonte, MG, Brasil

**Keywords:** Drug Utilization, Pharmacoepidemiology, Pharmaceutical Services, Primary Health Care, Health Services Research, Unified Health System, Uso de Medicamentos, Farmacoepidemiologia, Assistência Farmacêutica, Atenção Primária à Saúde, Pesquisa sobre Serviços de Saúde, Sistema Único de Saúde

## Abstract

**OBJECTIVE:**

To characterize the use of medicines by patients of the primary health care of the Brazilian Unified Health System (SUS).

**METHODS:**

This is a cross-sectional, exploratory, and descriptive study, part of the *Pesquisa Nacional sobre Acesso, Utilização e Promoção do Uso Racional de Medicamentos – Serviços, 2015* (PNAUM – National Survey on Access, Use and Promotion of Rational Use of Medicines – Services, 2015). Interviews were carried out with patients present in the services by semi-structured questionnaires. Sociodemographic, clinical, and use of medicines variables were assessed and the use of medicines in the 30 days prior to the interview was also verified. The population was stratified into three age groups: 18 to 44, 45 to 64, and 65 years or more. The differences between the age groups were verified using the Student’s t-test for continuous variables and chi-square test for the categorical ones. The complex samples analysis plan was employed. The medicines were classified according to the Anatomical Therapeutic Chemical Classification System.

**RESULTS:**

Of the 8,803 patients interviewed, 6,511 (76.2%) reported to have used medicines in the 30 days prior to the interview. On average, each patient used 2.32 medicines, without difference between the sexes. Among medicine users, 18.2% were aged 65 years or more. Compared to the other age groups, older adults presented more comorbidities, used more medicines, and self-reported worse health conditions. They were also less educated, reported worse economic situation, and lived alone. The medicines that were mostly used were “other analgesics and antipyretics” (3^rd^ ATC level) and Losartan (5^th^ ATC level).

**CONCLUSIONS:**

Most medicine users had lower education level and presented comorbidities. The most used medicines were the antihypertensive ones. Self-medication was higher among young people. Most patients reported to use generic medicines. The average number of medicines and the prevalence of use increased with age. Due to the characteristics observed and the difficulties in the use of medicines, older adults are in a situation of greater vulnerability.

## RESUMO

**OBJETIVO:**

Caracterizar o perfil de utilização de medicamentos pelos usuários da Atenção Primária do Sistema Único de Saúde no Brasil.

**MÉTODOS:**

Estudo transversal, exploratório, de natureza descritiva, integrante da Pesquisa Nacional Sobre Acesso, Utilização e Promoção do Uso Racional de Medicamentos – Serviços, 2015. Foram realizadas entrevistas com usuários presentes nos serviços por meio de questionários semiestruturados. Foram avaliadas as variáveis sociodemográficas, clínicas e relacionadas ao uso de medicamentos e verificado o uso de medicamentos nos 30 dias anteriores à entrevista. A população foi estratificada em três faixas etárias: 18 a 44, 45 a 64 e 65 anos ou mais. As diferenças entre as faixas etárias foram verificadas por meio do teste t de *Student* para variáveis contínuas e teste qui-quadrado para categóricas. Utilizou-se o plano de análises de amostras complexas. Os medicamentos foram classificados conforme *Anatomical Therapeutical Chemical Classification System*.

**RESULTADOS:**

De 8.803 usuários entrevistados, 6.511 (76,2%) relataram uso de medicamentos nos 30 dias anteriores à entrevista. Em média, cada usuário utilizou 2,32 medicamentos, sem diferença entre os sexos. Dentre os usuários de medicamentos, 18,2% tinham 65 anos de idade ou mais. Em comparação com as demais faixas etárias os idosos apresentaram mais comorbidades, usaram mais medicamentos e autorrelataram pior condição de saúde; eram menos escolarizados, relataram pior situação econômica e viviam sozinhos. Os medicamentos mais utilizados foram “outros analgésicos e antipiréticos” (3º nível ATC) e losartana (5º nível ATC).

**CONCLUSÕES:**

A maioria dos usuários de medicamentos possuía baixa escolaridade e comorbidades. Os medicamentos mais utilizados foram os anti-hipertensivos. A automedicação foi maior entre os jovens. A maioria dos usuários relatou utilização de medicamentos genéricos. O número médio de medicamentos e a prevalência de uso aumentaram com a idade. Devido às características observadas e as dificuldades no uso de medicamentos, os idosos estão em situação de maior vulnerabilidade.

## INTRODUCTION

Medicines have assumed an important role in reducing human suffering. They produce healing, prolong life, and delay the emergence of complications associated with the disease, thus facilitating the coexistence of the individual with his/her illness. Furthermore, medicines are considered highly cost-effective technologies and their proper use can influence the process of health care^20^.

The use of medicines is influenced by the demographic structure, socioeconomic, behavioral, and cultural factors, morbidity profile, characteristics of the pharmaceutical market, and Government policies directed to the sector^16^. The wide range of products, the pharmaceutical industry marketing, and the number of prescribed medicines are factors that can compromise the quality of the use of medicines^18^.

The increased prevalence of chronic diseases in the Country, especially arterial hypertension, diabetes, arthritis/osteoarthritis, and depression is a result of the rapid and growing process of ageing of the Brazilian population in recent years. In parallel with this process, the use of medicines is growing, necessary to control and prevent problems related to the health of individuals^19^.

According to data from the World Health Organization (WHO) over 50% of the medicines are prescribed or dispensed inappropriately all around the world, and about 50% of patients use medicines incorrectly, leading to high indexes of morbidity and mortality. The misuse of medicines is also related to the use of multiple medicines, inappropriate use of antibiotics and injectable medicines, self-medication, and prescriptions that do not comply with clinical guidelines^a^.

Primary health care (PHC), as part and coordinator of a health care network, must be prepared to solve almost all of the most common problems that arise in the context of primary health care. Completeness means the provision, by the health team, of a set of services that meet the needs of the population in the fields of promotion, prevention, cure, care, and rehabilitation. Access to medicines with quality and the promotion of their correct and timely use contributes to a resolutive PHC^14^.

The importance of medicines in health care is growing, either from an economic point of view or from the health point of view. The expansion of the population’s access to health care, by SUS, demanded, over the past years, changes in the organization of public Pharmaceutical Services (PS), to increase the coverage of free distribution of medicines, as well as the construction of a legal framework to support the process of decentralization of the management of PS actions^15^.

From the analysis of the consumption of medicines and of PS, it is possible to qualify the use of medicines, improving individual and collective health conditions, as well as deploying preventive or healing actions^9^.

The *Pesquisa Nacional sobre Acesso, Utilização e Uso Racional de Medicamentos – Serviços* (PNAUM – National Survey on Access, Use and Promotion of Rational Use of Medicines – Services) aimed at characterizing the coordination of pharmaceutical services in SUS primary health care to promote access and rational use of medicines, as well as identifying and discussing the factors that affect the consolidation of pharmaceutical services in the cities. In this context, this study aimed to characterize the profile of use of medicines by patients of SUS primary health care.

## METHODS

PNAUM is a cross-sectional, exploratory, evaluative study, consisting of information collection in a representative sample of primary health care services, in cities of the Brazilian regions. Face-to-face interviews were carried out with patients, physicians, and professionals responsible for the dispensing of medicines in the primary health care services of SUS. The study was comprised of various populations, with stratified samples by region. We opted for the use of sampling in various stages of selection, in each stage the populations were sampled and the estimates relating to them were made separately. Three samples were drawn: of cities, services, and patients. In the first, the cities were elements of the sample. In the second, these cities became primary sampling units, in which the services that composed the sample were drawn. In the third, the services became secondary sampling units, in which the patients (which are the focus of this study) were drawn. The PNAUM – Services methodology, as well as the sampling process, are described in detail by Álvares et al. (2016)^1^.

To characterize the profile of use of medicines, data from the interviews with the patients present in the PHC services were used. The use of medicines in the 30 days prior to the interview was verified. Sociodemographic, clinical, and medicine use (use of generic medicines, self-medication, need of help to use the medicines, medicines used, and use of the *Programa Farmácia Popular* (PFP – Popular Pharmacy Program)) variables were assessed.

The medicines were classified in therapeutic categories, by their active ingredient, according to the Anatomical Therapeutic Chemical Classification System (ATC). In this system, the substance is classified according to the organ or system on which it acts and to their chemical, pharmacological, and therapeutic properties. The medicines are divided in 14 main groups (1^st^ level), with pharmacological/therapeutic subgroups (2^nd^ level). The 3^rd^ and 4^th^ levels correspond to chemical/pharmacological/therapeutic subgroups, and the 5^th^ level to the chemical substance^26^.

Medicines of the following classes were classified as antihypertensive: antihypertensive (C02), diuretics (C03), beta blockers (C07), calcium channel blockers (C08), and agents acting in the renin-angiotensin system (C09). As antidiabetic, medicines used in diabetes (A10) were employed, and as antidepressants, the psycholeptics (N05) and psychoanaleptics (N06). Medicines for dyslipidemia were lipid modifiers agents (C10) and anti-obesity preparations, excluding dietetic products (A08). As there is no specific protocol for arthritis, osteoarthritis, and rheumatism, for these diseases the medicines considered were: corticosteroid medicines for simples systemic use (A07E), nonsteroidal and antirheumatic anti-inflammatory products (C01E), simple corticosteroids (D07A), corticosteroids for simple systemic use (H02A), immunosuppressants (L04A), nonsteroidal and antirheumatic anti-inflammatory products (M01A), other analgesics and antipyretics (N02B), and antimalarial medicines (P01B).

The relationship between the report of medical diagnosis of the most prevalent diseases and the use of medicines indicated for the treatment of these diseases was also assessed. The population was stratified into three age groups: 18 to 44, 45 to 64, and 65 years or more. The differences among the age groups were verified using the Student’s t-test for continuous variables and Pearson’s chi-square test for the categorical ones.

Data analysis was performed using the plan of complex samples of the SPSS® software version 22.

PNAUM was approved by the National Research Ethics Committee of the National Health Council, under Opinion no. 398,131/2013. All participants signed the informed consent form.

## RESULTS

A total of 8,803 patients were interviewed in 1,305 PHC services, located in 272 cities distributed in the five regions of Brazil. Of these, 6,511 patients (76.2%) reported having used medicines in the 30 days prior to the interview.

The average number of medicines used was 2.32 (95%CI: 2.186–2.554) per patient. The average for each age group varied significantly: 1.75 (95%CI: 1.65–1.85) for the age group of 18 to 44 years; 2.63 (95%CI: 2.45–2.81) for the 45 to 64 years age group; and 3.00 (95%CI: 2.74–3.25) for older adults (65 years or more). The prevalence of use of medicines increased according to the age group, being of 92.1% in persons aged 65 years or more.

There was a predominance of the female sex in the population that uses medicines, but the proportion of men increased as age increased, being 36.1% in the age group of 65 years or more. No differences were found in the use of medicines among the sexes. Most medicine users had some elementary school (43.2%) and only 3% reported to have a higher education degree. We observed the lowest education level among older adults, with a proportion of 26.5% of illiterate individuals. The majority of interviewed medicine users declared marital status married. Among older adults, 33.5% were widowers or divorced, with statistically significant differences compared to the other age groups. Regarding economic classification, most were from the economic class C, and, among the older adults, the classes D and E prevailed (Table 1).

**Table 1:** Sociodemographic and health characteristics of patients of the Primary Health Care of the Brazilian Unified Health System. National Survey on Access, Use and Promotion of Rational Use of Medicines – Services, 2015.

Variable	Age group (years)						p

	18 to 44		45 to 64		65 or more		
	n = 2,871		n = 2,499		n = 1,087		
		
	n*	% (95%CI)	n*	% (95%CI)	n*	% (95%CI)	
Sex							< 0.001
Female	2,369	82.1 (79.8–84.2)	1,890	75.8 (73.3–78.1)	719	63.9 (60.3–67.3)	
Male	502	17.9 (15.8–20.2)	609	24.2 (21.9–26.7)	368	36.1 (32.7–39.7)	
Marital status							< 0.001
Married/Common-law marriage	1,813	65.7 (63.1–68.3)	1,596	68.2 (65.4–70.8)	590	59.6 (55.1–63.9)	
Single	950	30.2 (28.0–32.6)	400	12.5 (10.8–14.3)	89	7.0 (5.3–9.0)	
Other	108	4.0 (3.2–5.1)	503	19.3 (17.1–21.8)	408	33.5 (29.6–37.6)	
Education level							< 0.001
Illiterate	71	3.2 (2.2–4.6)	293	12.9 (10.1–16.6)	278	26.5 (19.9–34.2)	
Some Elementary School	710	28.8 (26.0–31.8)	1,186	50.9 (46.7–55.0)	598	59.6 (50.8–67.8)	
Elementary School	731	24.3 (21.5–27.2)	467	17.9 (15.6–20.6)	110	8.6 (5.5–13.2)	
High School	1,237	39.3 (36.5–42.1)	474	16.1 (14.0–18.4)	81	3.8 (2.5–5.8)	
Higher Education	122	4.4 (3.4–5.8)	79	2.3 (1.6–3.2)	20	1.5 (0.8–2.8)	
Economic class							< 0.001
A and B	556	18.9 (15.9–22.2)	397	14.5 (12.1–17.4)	105	7.9 (5.8–10.5)	
C	1,803	59.1 (56.2–61.9)	1,417	53.7 (49.7–57.6)	545	45.9 (40.5–51.4)	
D and E	509	22.1 (18.3–26.4)	684	31.8 (26.5–37.6)	437	46.2 (39.6–52.9)	
Number of chronic diseases							< 0.001
None	1,309	43.6 (40.4–46.9)	213	7.9 (6.7–9.4)	34	3.1 (2.0–4.9)	
One	836	30.6 (28.1–33.2)	589	26.2 (23.1–29.5)	227	21.6 (18.2–25.3)	
Two or more	646	25.8 (22.9–28.9)	1,546	65.9 (62.1–69.4)	775	75.3 (71.0–79.2)	
Main chronic diseases							
Hypertension	551	22.0 (19.4–24.9)	1,513	62.3 (58.9–65.5)	872	81.7 (77.7–85.2)	< 0.001
Dyslipidemia	329	11.0 (9.5–12.8)	966	39.8 (36.6–43.1)	443	40.9 (36.9–45.1)	
Arthritis, osteoarthritis, or rheumatism	234	8.2 (6.7–10.1)	800	32.4 (28.7–36.3)	457	40.6 (35.4–46.2)	
Depression	456	19.0 (16.7–21.4)	665	26.7 (22.8–31.0)	216	19.9 (15.5–25.2)	
*Diabetes mellitus*	129	4.1 (3.1–5.4)	618	24.8 (22.3–27.6)	329	31.3 (27.2–35.8)	
Chronic lung disease	325	11.5 (9.8–13.6)	282	9.9 (8.2–11.8)	120	10.0 (7.4–13.4)	0.321
Heart diseases	103	3.6 (2.7–4.8)	269	11.1 (8.9–13.8)	224	20.8 (16.6–25.8)	< 0.001
Cerebrovascular accident	26	1.0 (0.6–1.6)	100	3.8 (2.8–5.2)	74	6.5 (4.5–9.4)	
Regions							< 0.001
North	608	6.8 (5.2–8.7)	344	4.1 (3.0–5.7)	126	3.0 (2.0–4.6)	
Northeast	552	31.9 (24.4–40.5)	443	25.7 (18.4–34.7)	207	28.8 (19.6–40.2)	
Midwest	500	6.3 (4.4–8.9)	408	5.0 (3.4–7.3)	180	4.8 (3.1–7.4)	
Southeast	564	29.4 (22.8–37.0)	597	36.4 (27.9–45.9)	249	36.9 (25.0–50.8)	
South	647	25.7 (19.3–33.3)	707	28.8 (21.4–37.6)	325	26.4 (17.9–37.3)	

Source: PNAUM – Services, 2015.

* Non-weighted n value

Most medicine users reported having one or more chronic diseases (77.6%), with a statistically significant difference among the age groups (p<0.001), and among the older adults the prevalence was of 96.9%. Among the major chronic diseases reported, hypertension was the most prevalent (48.9%). Hypertension, hyperlipidemia, arthritis/osteoarthritis or rheumatism, diabetes mellitus, heart disease, and stroke were more prevalent among older adults and the difference was significant among the age groups (p<0.001).

Regarding the need for help from another person to use the medicines, we observed greater proportions among older adults, 10% of which always need it and 4.4% sometimes.

Self-medication was higher among younger people (45.7%) with a statistically significant difference (p<0.001) per age group. Among the reasons for self-medication, the most reported were “previous use of the medicine” and “having it at home.”

When non-use of medicines prescribed by the physician was investigated, higher proportions were observed in users in the 18 to 44 years age group (16.7%). Among the reasons for this non-use stood out negative experience with the use of the same product (50.7%), believing to already be healed (49.3%) and considering that it was not necessary to use the medicine (46.1%). For the age group of 65 years or more, the main reasons for non-use of the medicine were believing that the medicine is not correct or does not work (49.4%) and previous negative experience (having used the medicine in the past and feeling sick because of it) (49.2%).

More than half (57.8%) of patients reported using generic medicines, but there was no significant difference between the age groups (Table 2). Concerning the Popular Pharmacy Program, the highest use was in the age group of 65 years or more (67.8%), followed by 45 to 64 years (63.8%) and 18 to 44 (49.0%).

**Table 2:** Characteristics of the use of medicines by patients of the Primary Health Care of the Brazilian Unified Health System. National Survey on Access. Use and Promotion of Rational Use of Medicines – Services, 2015.

Variable	Age group (years)						p

	18 to 44		45 to 64		65 or more		
	n = 2.871		n = 2.499		n = 1.087		
		
	n*	% (95%CI)	n*	% (95%CI)	n*	% (95%CI)	
Do you use a generic medicine?							0.375
Yes	1.606	55.8 (49.1–62.3)	1.518	58.1 (50.9–65.0)	714	62.0 (49.9–72.8)	
No	891	30.3 (23.9–37.5)	652	29.6 (22.3–38.2)	240	27.2 (16.2–41.9)	
Does not know	373	13.9 (10.3–18.5)	329	12.2 (9.3–16.0)	133	10.8 (7.5–15.2)	
Do you need help to use the medicines?							< 0.001
Always	90	4.2 (2.7–6.6)	94	4.7 (3.2–6.7)	94	10.0 (7.4–13.5)	
Sometimes	59	2.1 (1.4–3.2)	86	3.6 (2.5–5.1)	50	4.4 (3.0–6.6)	
No	2.721	93.6 (91.2–95.4)	2.318	91.8 (88.9–94.0)	943	85.5 (81.5–88.8)	
Use of medicines without prescription *(yes)*	1.392	45.7 (41.6–49.8)	930	36.5 (32.1–41.1)	283	27.8 (22.9–33.3)	< 0.001
Situations in which you use medicines without prescription							
Previous use	1.250	88.3 (84.2–91.5)	781	83.3 (76.8–88.3)	236	85.9 (79.1–90.8)	0.193
When you have the medicine at home	1.150	82.4 (73.7–88.7)	741	81.7 (72.1–88.5)	208	77.0 (62.4–87.1)	0.242
Indication in the pharmacy	1.037	71.4 (66.8–75.7)	577	60.8 (53.7–67.5)	156	51.8 (44.8–58.8)	< 0.001
Knows someone who has already used it	831	56.9 (51.2–62.4)	473	48.9 (42.5–55.5)	136	42.8 (34.9–51.0)	0.002
Easy access to the medicine	763	55.4 (47.7–62.9)	434	46.6 (38.6–54.8)	129	46.1 (33.8–58.9)	0.032
Read the consumer leaflet or other information	685	47.1 (42.3–52.0)	326	34.5 (28.7–40.7)	58	18.1 (12.5–25.4)	< 0.001
Ceases to use medicines prescribed by the physician *(yes)*	539	16.7 (14.3–19.4)	389	14.1 (11.6–17.0)	110	9.1 (6.2–13.1)	0.001
Cases in which you cease to use the prescribed medicines							
Used before and felt sick	326	50.7 (42.1–59.2)	224	47.9 (38.1–57.9)	62	49.2 (34.7–63.9)	0.741
Considers that it is not the right one or that it does not work	284	46.2 (38.2–54.4)	201	45.5 (36.7–54.6)	57	49.4 (37.7–61.2)	0.844
Thinks it is too strong or weak	276	44.3 (38.0–50.8)	164	39.0 (30.4–48.3)	40	32.0 (22.4–43.4)	0.396
Believes to be cured	282	49.3 (42.4–56.3)	152	40.2 (32.5–48.4)	39	34.9 (25.5–45.6)	0.046
Evaluates that it is not needed	267	46.1 (39.4–53.1)	160	40.7 (32.9–48.9)	39	32.4 (21.0–46.3)	0.043
When you read something considered bad in the consumer leaflet	175	26.4 (20.9–32.7)	96	21.4 (16.2–27.7)	17	9.2 (4.2–19.1)	0.002
Uses the Brazilian Popular Pharmacy Program *(yes)*	891	49.0 (42.2–55.8)	1.202	63.8 (58.9–68.4)	561	67.8 (59.4–75.1)	< 0.001

Source: PNAUM – Services, 2015.

* Non-weighted n value

Of the 15,061 medicines reported by patients, it was possible to identify properly 13,515 items, which were classified considering the 5^th^ ATC level. Among the self-reported products, 1,546 (9.3%) were described inappropriately, such as “I do not remember the name,” “maleate,” “cancer bacteria,” “sulfuric acid.”

Table 3 presents the most used medicine groups, considering the 3^rd^ ATC level. Of the total of medicines, 1,249 (8.1%) were classified as “other analgesics and antipyretics”; 819 (5.6%), “hypoglycemic medicines, excluding insulin”; and 765 (5.5%), “nonsteroidal and antirheumatic anti-inflammatory products.”

**Table 3:** Medicines most used by patients of the Primary Health Care of the Brazilian Unified Health System, considering the 3rd Anatomical Therapeutic Chemical level. National Survey on Access, Use and Promotion of Rational Use of Medicines – Services, 2015.

Pharmacological subgroup (3^rd^ ATC level)	ATC Code	n*	%(95%CI)
Other analgesics and antipyretics	N02B	1,249	8.1 (6.8–9.7)
Hypoglycemic medicines, excluding insulin	A10B	819	5.6 (5.1–6.3)
Nonsteroidal and antirheumatic anti-inflammatory products	M01A	765	5.5 (4.9–6.3)
Angiotensin-converting enzyme inhibitors	C09A	824	5.2 (4.5–6.1)
Angiotensin II antagonists	C09C	756	4.9 (3.9–6.2)
Antidepressants	N06A	645	4.8 (4.3–5.5)
Hypolipidemic agents	C10A	658	4.7 (4.1–5.3)
Medicines for peptic ulcer and gastroesophageal reflux disease	A02B	640	4.4 (3.9–4.9)
Beta blockers agents	C07A	615	4.2 (3.6–5.0)
Low power diuretics	C03A	619	3.8 (3.2–4.6)
Antiepileptics	N03A	427	3.0 (2.6–3.5)
Angiotensin-converting enzyme inhibitors, associations	C09B	321	2.5 (1.9–3.3)
Centrally acting muscle relaxants	M03B	378	2.5 (2.0–3.0)
Hormonal contraceptives for systemic use	G03A	338	2.2 (1.7–2.9)
Antithrombotic agents	B01A	262	1.9 (1.5–2.3)
Thyroid preparations	H03A	256	1.8 (1.5–2.2)
Anxiolytics medicines	N05B	224	1.8 (1.4–2.3)
Iron preparations	B03A	337	1.8 (1.4–2.1)
Calcium channel selective blockers	C08C	223	1.3 (1.0–1.7)
Beta-lactams antibacterials, penicillins	J01C	215	1.3 (1.1–1.6)

Source: PNAUM – Services, 2015.

* Non-weighted n value

Regarding the classification in the 5^th^ ATC level, the most used were: Losartan (4.8%), simvastatin (4.1%), omeprazole (3.9%), hydrochlorothiazide (3.4%), and metformin (3.2%) (Table 4).

**Table 4:** Medicines most used by patients of the Primary Health Care of the Brazilian Unified Health System, considering the 5th Anatomical Therapeutic Chemical level. National Survey on Access, Use and Promotion of Rational Use of Medicines – Services, 2015.

Name of the medicine	ATC Code	n^a^	%(95%CI)
Does not know	DA^b^	1,544	9.3 (7.6–11.3)
Losartan	C09CA01	739	4.8 (3.8–6.1)
Simvastatin	C10AA01	576	4.1 (3.5–4.7)
Omeprazole	A02BC01	556	3.9 (3.5–4.4)
Hydrochlorothiazide	C03AA03	552	3.4 (2.8–4.3)
Metformin	A10BA02	494	3.2 (2.7–3.8)
Captopril	C09AA01	424	2.8 (2.2–3.6)
Paracetamol	N02BE01	439	2.8 (2.3–3.3)
Dipyrone	N02BB02	378	2.4 (1.7–3.3)
Captopril + diuretic	C09BA01	309	2.4 (1.8–3.2)
Enalapril	C09AA02	391	2.3 (1.9–2.8)
Atenolol	C07AB03	321	2.1 (1.7–2.8)
Ibuprofen	M01AE01	284	2.0 (1.5–2.8)
Fluoxetine	N06AB03	244	1.9 (1.5–2.4)
Levothyroxine	H03AA01	256	1.8 (1.5–2.2)
Clonazepam	N03AE01	217	1.7 (1.4–2.1)
Ferrous sulfate	B03AA07	321	1.7 (1.4–2.1)
Diclofenac	M01AB05	229	1.7 (1.4–2.0)
Glibenclamide	A10BB01	206	1.5 (1.2–1.9)
Acetylsalicylic acid	N02BA01	246	1.4 (1.0–2.0)
Acetylsalicylic acid (antithrombotic agent)	B01AC06	198	1.4 (1.0–1.9)

Source: PNAUM – Services, 2015.

^a^ Non-weighted n value; ^b^ Does not apply

Among the respondents who reported having chronic diseases, 77.7% of those with hypertension and 62.1% of those with diabetes used specific medicines for the disease. Among the patients with dyslipidemia, arthritis, osteoarthritis or rheumatism, and depression, the majority did not report use of specific medicines for these diseases (Table 5).

**Table 5:** Percentage of patients of the Primary Health Care of the Brazilian Unified Health System that reported having a disease versus use of medicine specific to the disease. National Survey on Access, Use and Promotion of Rational Use of Medicines – Services, 2015.

Disease	Report of use of specific medicines for the disease	

	Yes % (95%CI)	No % (95%CI)
Hypertension	77.7 (74.3–80.8)	22.3 (19.2–25.7)
Diabetes	62.1 (58.1–65.8)	37.9 (34.2–41.9)
Arthritis, osteoarthritis, rheumatism	33.1 (28.2–38.5)	66.9 (61.5–71.8)
Depression	32.9 (29.4–36.7)	67.1 (63.3–70.6)
Dyslipidemia	28.7 (24.3–33.5)	71.3 (66.5–75.7)

Source: PNAUM – Services, 2015.

## DISCUSSION

Studies of use of medicines enable better knowledge on the characteristics of their users and the identification of factors associated with consumption, contributing to qualify the use and rationalize health resources^19^. Studies with nationwide representative data are scarce^2^ ^,^ ^5^, most being restricted to local research^4^ ^,^ ^13^ ^,^ ^19^.

This study was conducted all over Brazil, with a representative sample of patients of PHC, which sought the service for medical care. This characteristic may have influenced some of the results found in this study.

The prevalence of the use of medicines in this study (76.1%) was higher than those of other studies conducted on PHC^4^ ^,^ ^9^ and population-based^5^ ^,^ ^7^ ^,^ ^10^.

The prevalence of the use of medicines in individuals in the age group of 65 years or more (92.1%) was higher than that found in studies conducted with older adults both in PHC^11^ and in population-based studies^20^ ^,^ ^23^.

The number of medicines used by patients of PHC ranged from one to 16, a result similar to that observed in other national studies in PHC^25^ and population-based^3^. The average number of medicines used by individual (2.32) was similar to that observed by the Pan American Health Organization (2005)^17^ and higher than in other Brazilian studies carried out with adults^4^ ^,^ ^7^ ^,^ ^10^ ^,^ ^16^, whose averages ranged from 1.81 to 2.1, and was also lower than the average found in a study carried out in the primary health care of a district in India (2.76)^6^.

The average number of medicines used increases according to the age group, rising to 3.0 in the group 65 years or more. These results corroborate other national and international studies^3^ ^,^ ^7^ ^,^ ^19^ ^,^ ^21^ and are below the average found by Silva et al. (2012)^23^ in a national sample of older adults (3.8) and the 3.7 identified by Rozenfeld et al. (2008)^20^ among retired individuals of Rio de Janeiro. These studies have also associated greater use of medicines with higher level of income. In this study the majority of the population was concentrated on classes C, D and E. The lower participation of people of classes A and B may have contributed to the lower number of used medicines.

The average number of medicines used is an important indicator, which enables the measurement of the degree of polypharmacy of the patient, a predictor factor of medicine interactions and adverse events^8^. The use of multiple medicines is common in older adults due to the higher prevalence of chronic diseases and clinical manifestations resulting from aging. The consumption of medicines in this age group requires greater care, because older adults present physiological changes that affect clinical pharmacokinetics and may generate toxic effects and adverse events^22^. Women typically have greater concern for their health, seeking the health services more frequently, in addition to the existence of several health programs developed for them^19^. In this study, women accounted for the majority of respondents. However, no significant differences were observed in the prevalence of use of medicines among men (74.5%) and women (76.0%), in contrast with findings of other studies in which women showed a higher prevalence of use of medicines than men^3^ ^,^ ^5^ ^,^ ^7^ ^,^ ^25^.

We observed a low educational level among medicine users, a result similar to that observed in another national study carried out in PHC^19^ and different from what was observed in other national population-based studies^3^ ^,^ ^7^ ^,^ ^20^. This correlation is troubling, since low educational level can affect the degree of understanding of the prescribed scheme and adherence to treatment. Such differences can be explained by the different characteristics of the evaluated populations.

Among medicine users, 77.6% had a chronic disease, a result similar to the 77% found by Carvalho (2005)^5^. People with chronic diseases seek the services more, and medicines are one of the most used therapeutic interventions^7^. The high rate of chronic diseases verified in the older adult population and the increased use of medicines in this age group reinforce these findings.

The self-report of use of generic medicines was registered by 57.8% of medicine users. This data can be due to the change in the legislation that regulates public procurement in Brazil. According to the National Policy of Medicines, promoting the use of generic medicines is a priority guideline. However, despite the compulsory adoption of the generic designation in public purchases and bidding of medicines performed by the Public Administration, according to law 8,666/93, management instances of SUS must purchase medicines at the lowest price, after the technical requirements of the notice are fulfilled. Thus, the medicines provided by SUS may or may not be generic.

When assessing the most used pharmacological groups, painkillers and antipyretics stood out as the main ones, consistent with other national and international studies^3^ ^,^ ^5^ ^,^ ^21^. However, when assessed in isolation, it is possible to observe that the medicines that act on the cardiovascular system, particularly antihypertensives, are the most used group. These data are consistent with the demographic transition process experienced by the Country, where the growth of non-communicable chronic diseases is associated with the occurrence of acute conditions still relevant to the health care^14^.

When assessing the 20 most used medicines, seven of them belong to group C (medicines for the cardiovascular system) and two are antidiabetic (A10 group). There is coherence between the profile of use and the chronic diseases self-referred by patients of PHC of SUS. Like other national studies^7^ ^,^ ^10^ ^,^ ^20^ ^,^ ^23^, hypertension was the most reported disease. This result is consistent with the Brazilian epidemiological profile, in which cardiovascular diseases have high prevalence, including hypertension, especially in the population aged over 65 years^5^ ^,^ ^13^.

The most used medicine by the population of the study was Losartan (4.8%), blocker of the AT1 receptor of angiotensin II. In other national studies^7^ ^,^ ^16^, antihypertensives were also the most used medicines, especially the hydrochlorothiazide diuretic. According to the Brazilian Guidelines of Hypertension^24^ in effect, the pharmacological treatment of hypertension begins with monotherapy, with the AT1 receptor of angiotensin II blocker usually being the prescribed medicine. In the treatment of hypertension, especially in high-risk populations or with cardiovascular comorbidities, AT1 receptor of angiotensin II blockers provide reduction of cardiovascular morbidity and mortality and have a cerebrovascular protective effect superior to other antihypertensives, justifying the growing prescription of this pharmacological class.

We highlight that all the 20 most used medicines by patients of PHC belong to the National List of Medicines in force, being financed by the Basic Component of Pharmaceutical Services and/or provided by the Popular Pharmacy Program. This result suggests that prescribers have used the public medicines lists as guides for prescription within SUS, favoring free access to essential medicines.

Among medicine users, a significant portion (9.3%) did not know which medicine they were using or for what disease it was prescribed. Although some users reported the reason for which they were using the medicine, as for example “for high blood pressure” or “anti-inflammatory,” a large number of people responded “I do not know” or “I do not remember.” The low educational level identified especially among older adults (26.5%), age group that most uses medicines in this study, can justify the results found. This ignorance is an important finding, indicating the need for the development of continuous health education strategies, by the multi-professional teams, to contribute to the correct use of medicines.

The prevalence of referred self-medication (38.8%) was higher than in other national studies^2^ ^,^ ^12^, being higher in the age group from 18 to 44 years. Economic, political, and cultural factors have contributed to the growth and spread of self-medication in the world, making it a public health problem. According to Arrais (1997)^2^, the inadequate supply of medicines and the non-execution of compulsory submission of medical prescriptions, as well as low education levels, are the most cited reasons for the high frequency of self-medication in Brazil. In this study, previous satisfactory experience with the medicine, the availability of the product at home, and indication by pharmacy professionals were the main reasons for the use of nonprescription medicines.

Regarding the Popular Pharmacy Program, we observed that obtaining medicines from the program is higher as the age group increases. These data are consistent with the profile of medicines provided for free^b^ by drugstores and pharmacies accredited in the Popular Pharmacy Program, which include medicines for the treatment of chronic non-communicable diseases. Hypertension and diabetes, diseases whose treatments are listed as of free provision of medicines through the Popular Pharmacy Program, were the most referred to by the population aged over 65 years.

In this study, only 10% of older adults declared they needed help to use the medicines. In general, we also noted that most respondents did not need help to use the medicines, which was also observed in another study with older adults^20^.

This study showed that 78% of hypertensive respondents used medicines indicated for hypertension, and about 62% of diabetics used medicines for diabetes. The percentage of individuals who did not use medicines for those diseases can be explained by the deployment of the desirable non-pharmacological interventions for the treatment of these diseases.

However, for dyslipidemia, arthritis, osteoarthritis and rheumatism, and depression, 28.7%, 33.1%, and 32.9%, respectively, of respondents who reported having these comorbidities used specific medicines to treat them.

One of the limitations of this study is that, being a cross-sectional study, it does not allow the identification of the cause and effect relationship. In addition, a recall period of 30 days was used to evaluate the use of medicines. This criterion may have resulted in some memory bias, which becomes more pronounced the longer the period to be remembered, the age, and the number of medicines used in the period. Patients with hindered access to public health units are not represented as the usage data were obtained from interviews with patients of UBS.

In conclusion, the profile of medicine users verified in this study was composed predominantly by people with low education level and with comorbidities, associated with the identification of a significant percentage of people who did not know the name of the medicines they used. The most used medicines were the antihypertensive ones. Self-medication was higher among young people. Most patients reported to use generic medicines. The average number of medicines and the prevalence of use increased with age. Older patients require special attention and specific actions, as they presented low education level, had less access to consumer goods, reported the presence of more comorbidities and, when compared to other groups, reported difficulties in the use of medicines, which may put them in a situation of greater vulnerability.

## Footnotes

a World Health Organization, Quality Assurance and Safety of Medicines Team. The safety of medicines in public health programmes: pharmacovigilance an essential tool. Geneva: WHO; 2006 [cited 2016 Mar 4]. Available from: http://apps.who.int/iris/bitstream/10665/43384 /1/9241593911_eng.pdf

b Medicines provided freely by the Popular Pharmacy Program comprise the strategy “Saúde não tem preço,” which includes medicines for hypertension, diabetes, and asthma. Ministério da Saúde (BR), Portal Saúde. Elenco oficial dos medicamentos disponibilizados gratuitamente pelo Programa Aqui tem Farmácia Popular (updated on 01/29/2016). Brasília (DF); 2016 [cited 2016 Mar 4]. Available from: http://portalsaude.saude.gov.br/images /pdf/2016/fevereiro/02/rol-medicamentos-SNTP-290116.pdf
